# *Mp*ADC, an l-aspartate-α-decarboxylase, from *Myzus persicae,* that enables production of β-alanine with high yield by whole-cell enzymatic catalysis

**DOI:** 10.1186/s13068-023-02405-0

**Published:** 2023-10-24

**Authors:** Pengfu Liu, Saixue Xie, Qian Guo, Yan Chen, Junying Fan, Ashok Kumar Nadda, Xiaoluo Huang, Xiaohe Chu

**Affiliations:** 1 Collaborative Innovation Center of Yangtze River Delta Region Green Pharmaceuticals, Zhejiang University of Technology, Hangzhou, 310014 Zhejiang People’s Republic of China; 2 Shenzhen Institute of Synthetic Biology, Shenzhen Institute of Advanced Technology, Chinese Academy of Sciences, Shenzhen, 518055 Guangdong People’s Republic of China; 3 Department of Biotechnology and Bioinformatics, Jaypee University of Information Technology, 173234, Waknaghat, Solan, Himachal Pradesh India

**Keywords:** β-Alanine, l-Aspartate-α-decarboxylase, *Mp*ADC, Three-dimensional structure prediction, Whole-cell transformation

## Abstract

**Background:**

β-Alanine is a precursor of many important pharmaceutical products and food additives, its market demand is continuously increasing nowadays. Whole-cell catalysis relying on the recombinant expression of key β-alanine synthesizing enzymes is an important method to produce β-alanine. Nevertheless, β-alanine synthesizing enzymes found so far have problems including easy inactivation, low expression or poor catalytic activity, and it remains necessary to develop new enzymes.

**Results:**

Herein, we characterized an l-aspartate-α-decarboxylase, *Mp*ADC, from an aphid, *Myzus persicae*. It showed excellent catalytic activity at pH 6.0–7.5 and 37 °C. With the help of chaperone co-expression and N-terminal engineering guided by AlphaFold2 structure prediction, the expression and catalytic ability of *Mp*ADC in *Escherichia coli* were significantly improved. Using 50 g/L of *E. coli* cells expressing the *Mp*ADC-∆39 variant cultured in a 15-L fermenter, 232.36 g/L of β-alanine was synthesized in 13.5 h, with the average β-alanine yield of 17.22 g/L/h, which is best known so far.

**Conclusions:**

Our research should facilitate the production of β-alanine in an environment-friendly manner.

## Introduction

β-Alanine, also known as 3-aminopropionic acid, is the only naturally occurring β-amino acid and has an important function in organism’s metabolism [1]. β-alanine and its derivatives have been applied widely in pharmaceutical, feed and food industries. For example, it has been used as an intermediate for synthesis of disodium pamidronate [2], calcium pantothenate and carnosine [3], and also serves as sport supplements [4].

Many chemical methods, including the acrylonitrile method, and acrylic acid method, have been applied in the industrial production of β-alanine [5–7]. These methods exhibit limitations, such as high cost, high energy consumption, and serious environmental pollution [8]. Several biological methods including microbial fermentation [9, 10], enzyme catalysis [11, 12] and whole-cell transformation [8, 13] have drawn much attention in recent years due to their advantages. While microbial fermentation requires genetically engineered microorganism, optimized conditions, and enzyme catalysis in vitro necessitates the purification of the enzyme with a relatively high cost [14]. Thus, whole-cell transformation has become the most promising strategy to produce β-alanine owing to its simple work flow, environmental friendliness and potential low cost [15].

The biological methods to produce β-alanine rely on a key l-aspartate-α-decarboxylase (ADC) which catalyzes the decarboxylation of l-aspartate (L-ASP) [16–18]. There are two main types of ADC have been isolated and used in previous studies. The first type of ADC exists in bacteria in the homotetramer state, with pyruvyl group as a cofactor [19]. The other type of ADC is mainly isolated from insects and exists as a homodimer with pyridoxal-5′-phosphate (PLP) as a cofactor [20, 21]. The amino acid sequence and three-dimensional structure of ADCs from bacteria and insects are quite different, indicating that they evolved independently [17]. The insect ADCs share a high degree of sequence identity with cysteine sulfuric acid decarboxylase (CSADC) from mammalian and have also been annotated as CSADC in some databases [22–24].

ADCs from bacteria including *Bacillus subtilis*, *Escherichia coli* and *Corynebacterium glutamicum* have been well studied and used for β-alanine synthesis in the laboratory [9, 12, 20, 25]. For example, by expressing ADC from *C. glutamicum* in *E. coli* for whole-cell catalysis, β-alanine production reached 24.8 g/L with a yield of 92.6% after 20 h of reaction [25]. Moreover, the yield of β-alanine production reaches 215.30 g/L by a whole-cell catalysis reaction of 9 h using *E. coli* cells expressing E56S mutant from *B. subtilis* with OD_600_ of 100 [11]. Recently, the ADC from *Bacillus aryabhattai* Gel-09 (*Ba*ADC) was cloned and expressed. Through the fed-batch method using the variant *Ba*ADC_I88M, the catalytic conversion of L-ASP to β-alanine reached 98.6% (128.67 g/L) within 12 h [26]. Nevertheless, ADCs derived from bacteria show substrate-dependent inactivation. Bacterial ADCs perform autocatalytic intramolecular self-cleaving reactions on conserved serine residues and generate a pyruvyl moiety, which is essential for their catalytic activity in the L-ASP decarboxylation reaction [27]. For instance, the ADC from *E. coli* (also known as the PanD) is initially synthesized as a pro-enzyme (π-Chain, 13.8 kDa), and then it is subjected to self-shear processing at Gly24-Ser25 to generate an 11-kDa β-chain with a hydroxyl group at the C-terminus and a 2.8-kDa α-chain with a pyruvyl group at the N-terminus. It has been observed that the abnormal protonation of the imine structure during L-ASP decarboxylation reaction results in irreversible modification of active site and loss of the catalytic function [18, 27]. Therefore, large amounts of the bacterial ADC have to be invested into the reaction for the sake of replacing the inactivated ADC during bioconversion process, which are of low efficiency and high cost. A variant of *B. subtilis* ADC, *Bs*PanD_I46V/I88M/K104S/I126*_, reduced the chance of incorrect protonation and exhibited a 3.48-fold improved catalytic half-life compared to its wild-type [21]. However, it does not completely solve the problem of mechanism-based inactivation.

The structure and catalytic mechanism of insect ADCs are different from that of bacteria ADCs, with no mechanism-based inactivation effect, which enables it to become an excellent candidate for β-alanine synthesis. So far, several insect ADCs including that from *Aedes aegypti* (*Ae*ADC), *Drosophila melanogaster* (*Dm*ADC), and *Tribolium castaneum* (*Tc*ADC) have been investigated, yet only the *Tc*ADC, has been employed in the synthesis of β-alanine [17, 22, 28]. In 2019, Liu et al*.* expressed a cysteine sulfinic acid decarboxylase from *T. castaneum* (*Tc*CSADC) in *E. coli* and showed that *Tc*CSADC was able to catalyze the formation of β-alanine from aspartate and showed much higher specific activity than that of ADCs from prokaryotes. Moreover, 162 g/L of β-alanine could be produced by 35 h of whole-cell catalysis using cells with OD_600_ of 200 expressing its mutant, G369A [23]. Notably, they were mainly expressed as the inclusion bodies in *E. coli* and showed quite low cellular activity, which should strongly increase the catalytic cell investment and the overall cost of whole-cell catalysis, and limited their further large-scaled application. Therefore, it remains necessary to look for new ADCs with highly soluble expression and better catalytic efficiency to achieve high-yield of β-alanine using whole-cell catalysis.

In this study, we characterized an ADC, *Mp*ADC, from *Myzus persicae*. With the aid of chaperone protein expression, a considerable amount of *Mp*ADC was expressed in the soluble state. Moreover, truncation of the N-terminal 39 (*Mp*ADC-∆39 mutant) apparently increased soluble protein expression compared to the wild-type *Mp*ADC. Through established process of whole-cell enzymatic catalysis with *E. coli* cell expressing *Mp*ADC-∆39, high yield of β-alanine was obtained with small amounts of bacteria. Our study should provide a very good basis for the industrial production of β-alanine by whole-cell bioconversion.

## Materials and methods

### Chemicals and reagents

The restriction enzymes, marker DNA, and other reagents for gene manipulations were purchased from Thermo Fisher Scientific CN (Shanghai, China). HisTrapTMFF column and Sephadex^TM^ G-25M gel filtration column were purchased from GE® Healthcare company (Shanghai, China).

### Bioinformatic characterization of *Mp*ADC

ADC candidates were searched from the NCBI database by PSI-BLAST (https://blast.ncbi.nlm.nih.gov/Blast.cgi). The phylogenetic tree was constructed by the MEGA7 program [29]. The sequence conservation was evaluated using Clustal Omega (https://www.ebi.ac.uk/Tools/msa/clustalo/) and ENDscript 3.0 (https://espript.ibcp.fr/ESPript/ESPript/) [30].

### Recombinant *Mp*ADC expression and optimization

In order to increase the expression of *Mp*ADC*,* it was firstly codon-optimized, and synthesized into the *Nde*I and *Bam*HI sites of pET-28a (+) vector by GenScript (Nanjing, China), resulting a recombinant expressed *Mp*ADC with a His-tag at its N-terminal. The recombinant plasmid was then transformed into *E. coli* BL21 (DE3). To evaluate the protein expression, the recombinant strain was inoculated in 5 mL LB medium at 37 °C. After 12 h, the seed culture was transferred into 200 mL TB medium. When its OD_600_ reached 0.8–1.2, isopropyl-β-d-thiogalactopyranoside (IPTG) was added into the culture to a final concentration of 0.2 mM. After further incubation for 20 h at 20 °C, cells were collected by centrifugation at 4 °C, 9000×*g* for 10 min. Further, the harvested cells were resuspended in 50 mM PBS buffer (pH 7.0) and lysed with an ultrasonic oscillator. The cellular debris was removed by centrifugation at 4 °C, 10,000×*g* for 30 min, and the supernatant and pellet were used for protein expression assessment by SDS-PAGE. Notably, to enhance *Mp*ADC expression, plasmids pGrO7, pG-KJE8, pTf16, pKJE7 and pGTf2 expressing the chaperone protein derivatives were transformed into the *E. coli* BL21 (DE3) harboring pET28a-*MpADC* and its expression was evaluated using the same procedure as described above [31–33].

### *Mp*ADC purification and characterization

The supernatant of expressed *Mp*ADC was used for protein purification by one-step Ni^2+^-NTA affinity purification. The spectra of *Mp*ADC were obtained by an Agilent Bio Tek Synergy LX Multi-functional microplate detector. The target protein was collected for further activity assay. The protein concentration was determined by the Bradford method using bovine serum albumin as a standard [34]. The standard enzyme activity was assayed in a reaction mixture containing 920 μL reaction buffer (0.2 M phosphate buffer, pH 7.0), 10 μL 100 g/L L-ASP solution (adjusted to pH 7.0 with NaOH), 40 μL 0.05 M PLP, and 10 μL purified enzyme (approximately 45 μg). After incubation at 37 °C, 1000 rpm for 10 min, the reaction was stopped by adding 20 μL SDS (10%). Samples were treated with *o*-phthalaldehyde for derivatization as described previously [35] and analyzed by high-performance liquid chromatography (HPLC) (Agilent, Palo Alto, CA, USA) using a C18 column (Welch Ultimate® AQ-C18, 4.6 × 250 mm, 5 μm) maintained at 35 °C with the mobile phase containing 50 mM NaAc buffer (45%) at pH 3.5 and Methanol (55%). One unit of ADC activity was defined as the amount of enzyme that catalyzes the reaction to produce 1 μmol of β-alanine per minute under the described conditions.

To determine the effects of pH and temperature on enzyme activity, the reactions were performed under different conditions. Acetic acid buffer (pH 4.0–5.5), phosphate buffer (pH 5.5–7.5) and Tris–HCl buffer (pH7.5–9.0) were used to determine the optimal pH of *Mp*ADC. To investigate pH stability, the same buffer was used. The purified enzymes were incubated in the buffer system at 30 °C for 12 h, then the residual activity of ADC was determined at pH 7.5. To determine the optimum temperature, the enzyme activity of ADC was measured at 20 °C, 25 °C, 30 °C, 35 °C, 37 °C, 40 °C, 45 °C, 50 °C and 60 °C, respectively. For thermostability, the pure enzyme was incubated at different temperatures, including -20 °C, 4 °C, 15 °C, 25 °C, 30 °C, 35 °C, 37 °C, 50 °C and 60 °C for 12 h, and then the activity was measured at 37 °C. The activity of incubated at -20 °C for 12 h was defined as 100%. In order to investigate the optimal concentration of PLP for ADC, different concentrations of PLP were added into the reaction mixture and relative activities were tested. To value the effects of different metal ions toward the enzyme, 1 mM of Ca^2+^, Mn^2+^, Mg^2+^, Cu^2+^, Fe^2+^, Fe^3+^, Ni^2+^, Zn^2+^, Co^2+^ were, respectively, added into the standard enzymatic reaction and the relative activities were measured.

### Optimization of the whole-cell catalysis reaction by *E. coli* expressing *Mp*ADC

The reaction environment usually has different effects on the enzyme encapsulated in cells and those exposed to solution. To evaluate the effects of pH and temperature to cell expressing *MpADC* enzyme, the relative activity was detected under different pHs and temperature, respectively.

To investigate the effects of different concentrations of substrate to catalytic efficiency, 1 mL of the reaction mixture consisted of 50 g/L wet cell expressing ADC (the corresponding OD_600_ was measured at 18.4), 0.5 mM PLP, 200 mM phosphate buffer (pH = 6.5) and sodium L-ASP in different final concentration including 0, 5, 10, 20, 40, 60, 80, 100 and 120 g/L. After incubation for 5 min at 37 °C, the mixture was centrifuged at 11,000×*g* for 2 min and β-alanine content in the supernatant was measured.

Then, 1000 mL of reaction mixture containing 50 g/L wet cells expressing ADC, 0.5 mM PLP, 200 mM phosphate buffer (pH 6.0) and sodium L-ASP in different final concentrations including 10, 60 and 120 g/L. The mixtures were stirred with a magnetic stirrer (IKA® C-MAG HS 7) at 1000 rpm, 37 °C. The L-ASP powder was gradually added to maintain pH in the 6.0–6.5 range and in three different concentration ranges of 0–10, 10–60 and 60–110 g/L. Samples were taken at 1-h intervals, then L-ASP and alanine concentrations were measured by HPLC.

### Structure prediction of *Mp*ADC and analysis

The 3D structure of *Mp*ADC was modeled by Colabfold implemented under AlphaFold2 by selection of “non-template” and “unpaired + paired” MSA, with 6 rounds of iteration (https://colab.research.google.com/github/sokrypton/ColabFold/blob/main/AlphaFold2.iPynb) [36]. Five structural models obtained were then subjected to structure assessment by SAVESv6.0 (https://saves.mbi.ucla.edu/) [37–39]. Further, AutoDock 4.2.6 was used for molecular docking and PyMoL was used for the results visualization.

### N-terminal truncation of *Mp*ADC

The N-terminal peptide containing amino acids 1–39 was truncated by PCR. Designated forward and reverse primers (5′-ctggtgccgcgcggcagccatatggagagcctgagcgcgg-3′ and 5′-atggctgccgcgcggcaccaggccgctgctgtgatgatgatgat-3′**)** were used to amplify the template pET-28a*-MpADC* by *KOD-Plus-Neo* polymerase (TOYOBO, Japan) by the following procedure: 94 °C, 2 min; 98 °C, 10 s, 65 °C, 30 s, 68 °C, 4 min, 30 cycles; 68 °C, 5 min and 16 °C, 5 min.

### Fed-batch cultivation and whole-cell catalysis reaction

A single colony of the recombinant strain was inoculated into 5 mL LB medium supplemented with 50 μg/mL kanamycin and 25 μg/mL chloramphenicol, and after its incubation at 37 °C, 220 rpm for 10 h, it was transferred into 500 mL TB medium supplemented with kanamycin and chloramphenicol for further incubation at 37 °C, 220 rpm for 8 h. TB culture was then transferred into 10 L TB medium supplemented with 50 μg/mL kanamycin, 25 μg/mL chloramphenicol and 0.5 g/L arabinose, and fermented in a 15-L fermentor (Shanghai BaoXing Bio-Engineering Equipment Co., Ltd). After fermentation at 37 °C, 220 rpm for 5 h, additional feed containing 40% glycerol, 3% peptone and 1% yeast extract was added into the fermenter at a speed of 75 mL/h. pH was adjusted to by aqua ammonia. While OD_600_ of the fermented cell reached about 25, the temperature dropped to 20 °C and IPTG was added into the culture to a final concentration of 0.2 mM. After further incubation for around 18 h, cells were harvested and frozen at − 80 °C.

The whole-cell catalysis was performed in a 1-L reaction system containing 50 g fermented cells, 55 g/L L-ASP, 0.5 mM PLP (pH–6.3) on a magnetic stirrer (IKA® C-MAG HS 7) at 1500 rpm, 37 °C. pH of the reaction was kept at range of 6.0–6.5 by continuously adding solid powder of L-ASP. When pH changes slowed down after about 12 h, 20% sulfuric acid was used instead of L-ASP to regulate pH until pH no longer changes. The consumption of L-ASP and the production of β-alanine were measured at specific intervals.

## Results

### In silico analysis of *Mp*ADC

In contrast to the PanDs from prokaryotes, insect ADCs do not present mechanism-based inactivation by protein self-processing and should have more potential for industrial application. However, up to now, only three ADCs from insects have been verified, which were derived from *T. castaneum* (*Tc*ADC, GenBank accession number NM_001102585.1), *D. melanogaster* (GenBank accession number NP_476788.1) and *A. aegypti* (*Ae*ADC, GenBank accession number XM_001658385.2), and the relatively low enzyme activity or soluble expression of them restrict their practical applications for large-scaled β-alanine production by whole-cell catalysis [17, 23]. To explore better ADC candidates, we searched for ADC homologues from NCBI protein database using *Tc*ADC and *Ae*ADC as templates. Through preliminary activity screening of candidates that expressed in *E. coli* (data not shown), *Mp*ADC (GenBank accession number XP_022171514.1, CSADC from *M. persicae*), with the identity of 66.67, 65.03% and 62.11% towards *Tc*ADC, *Ae*ADC and *Dm*ADC, was selected for further study owing to its good catalytic activity.

Insects ADCs have high sequence identity with mammalian cysteine sulfinic acid decarboxylases (CSADCs) and glutamate decarboxylases (GAD), and it has been suggested that *Ae*ADC and *Dm*ADC have CSADC activity [16, 24, 26]. *Mp*ADC is previously annotated as cysteine sulfinic acid decarboxylase in NCBI. In order to further study the evolutionary relationship between *Mp*ADC and other characterized CSADCs, enzymes defined with activity of cysteine sulfinic acid decarboxylase from the Uniprot database were employed for analysis.

**Fig. 1:**
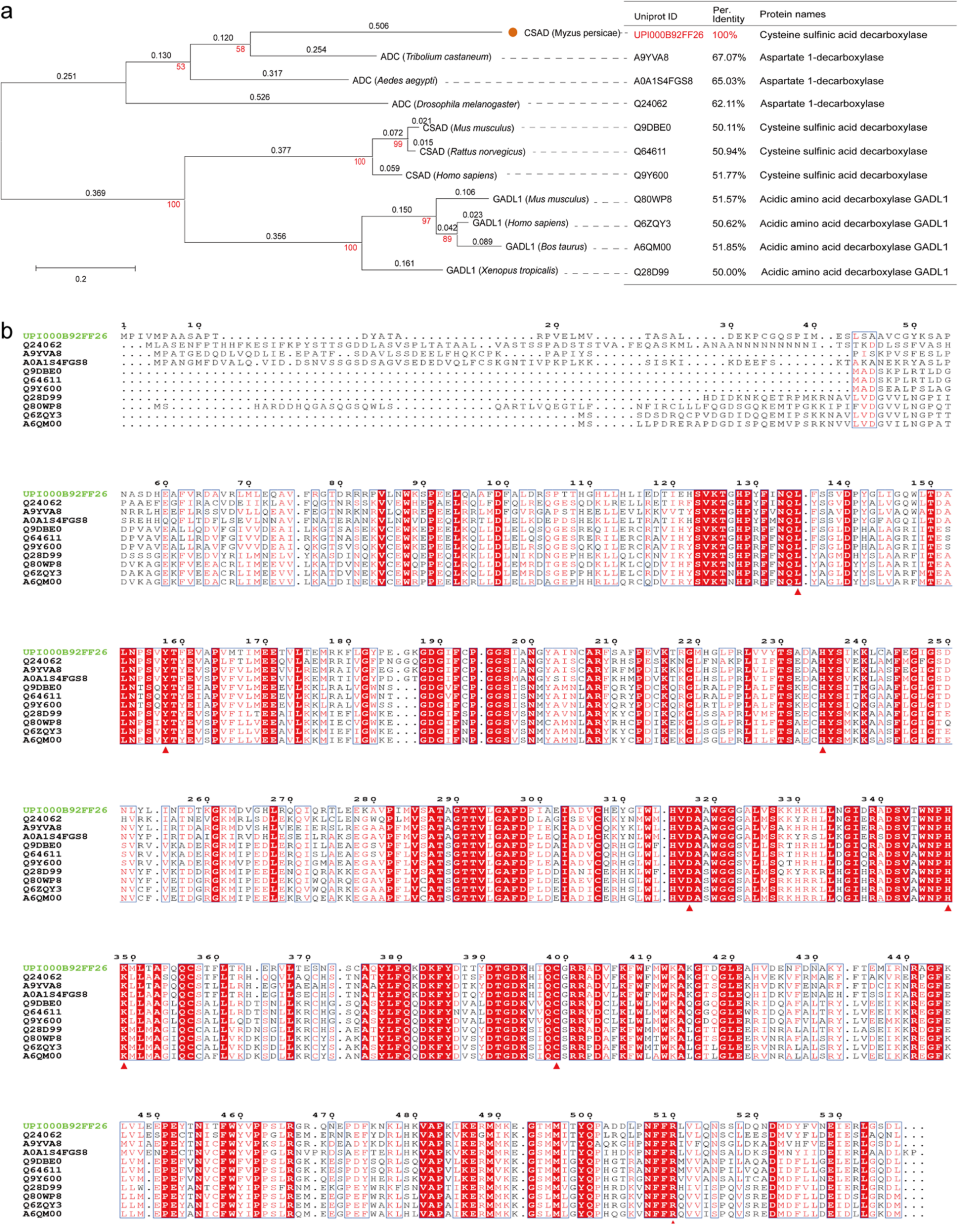
The phylogenetic and amino acid conservation analyses of *Mp*ADC. **a** Phylogenetic tree between *Mp*ADC and other ADCs. Maximum likelihood phylogenetic tree was constructed using MEGA7.0 program, with bootstrap values from 1000 replications. The red numerical character displayed on each branch represents the bootstrap value, and the black shows the evolutionary distance. The orange solid dots indicate *Mp*ADC. The sources of these enzymes were indicated inside parentheses. The Uniprot ID and sequence homology to *Mp*ADC of these enzymes were shown in the right part of the figure. **b** Multiple sequence alignment between *Mp*ADC and other ADCs. The conserved residues that were predicted to involve in the activity of *Mp*ADC were indicated with red triangles

Insect ADCs, mammalian CSADCs and GADL1s, are PLP-dependent decarboxylases (PLP-DC). Phylogenetic tree analysis shows that *Mp*ADC, *Tc*ADC, *Dm*ADC and *Ae*ADC form a group that is phylogenetically distant from those GADL1s and CSADs from the mammalian group. The *Mp*ADC also shows a relative phylogenetic distance from other insect ADCs. Moreover, the sequence identity between *Mp*ADC and alternative amino acid sequences ranged from 50 to 67.07% (Fig. 1a). Therefore, *Mp*ADC should be a novel member of ADC families that is distinct to current characterized CSADCs. Notably, multiple sequence alignments (Fig. 1b) indicated that the N-terminuses of these enzymes are poorly conserved compared to the C-terminus. The residues that generally interacted with PLP were also conserved in this group of decarboxylases (Fig. 1b), indicating its similar PLP binding mechanism [35, 40, 41].

### Expression optimization and purification of *Mp*ADC

**Fig. 2:**
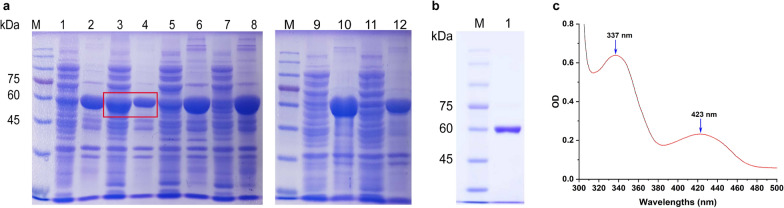
Analysis of *Mp*ADC expression and purified *Mp*ADC by SDS-PAGE and spectrometry. **a** SDS-PAGE analysis of protein expression in *E. coli* harboring pET-28a (+)-*Mp*ADC and different chaperone expression plasmids. Lane M: molecular weight protein standard; lanes 1, 3, 5, 7, 9, 11: the expression supernatant from the disrupted *E.coli* cell, which harboring pET-28a (+)-*Mp*ADC, pET-28a(+)-*Mp*ADC/pGrO7, pET-28a(+)-*Mp*ADC/pG-KJE8, pET-28a(+)-*Mp*ADC/pTf16, pET-28a(+)-*Mp*ADC/pKJE7, pET-28a(+)-*Mp*ADC/pGTf2, respectively; lanes 2, 4, 6, 8, 10, 12: the expression precipitation from the disrupted *E. coil cell*, harboring the plasmids with order above. The red box indicates the combination with highest soluble expression of *Mp*ADC. **b** SDS-PAGE analysis of purified *Mp*ADC. Lane M, molecular weight protein standard; lane 1: purified *Mp*ADC protein. **c** UV–visible spectra of the purified *Mp*ADC in 50 mM phosphate buffer (pH 7.0). The UV/visible spectrum from 300 to 500 nm was determined using an Agilent Bio Tek Synergy LX Multi-functional microplate detector. The presence of absorption peak with a *λ*max at 337 and 423 nm indicated by blue arrow

*Mp*ADC gene fragment was inserted into pET28a vector and then transformed into *E. coli* BL21 (DE3). Similar to other insect ADCs, *Mp*ADC was still mostly expressed as inclusion body under normal culture conditions. In order to enhance soluble expression of *Mp*ADC, five plasmids carrying different chaperone proteins including pGrO7, pG-KJE8, pTf16, pKJE7, and pGTf2 were introduced to the *E. coli* cells harboring pET28a-*Mp*ADC separately. By induction at 20 °C, *Mp*ADC expressed mainly in the supernatant of the host harboring pGrO7 (Fig. 2a), suggesting that *Mp*ADC was successfully expressed as soluble state with the help of chaperone proteins under low temperature. *Mp*ADC was purified to homogeneity by one-step Ni^2+^-NTA affinity chromatography for further study of enzymatic properties. As shown in Fig. 2b, *Mp*ADC exhibits a clear band at a molecular mass of approximately 60 kDa, corresponding to its predicted molecular mass.

When insect ADC sequences were compared to those of other PLP-dependent enzymes, it was discovered that the residues involved in PLP binding were conserved (Fig. 1b, the residue in *Mp*ADC is Lys349) [42]. It has been shown that PLP is typically connected with this type of enzymes via a conserved active-site lysine residue to create a Schiff base; as a result, the ionization status of the internal aldimine is influenced by residues in close proximity as well as residues that interact with the cofactor. All of these enzymes produce absorption bands with two Crest waves in the 320–500 nm wavelength region, corresponding to the enolimine and zwitterion forms of the internal aldimine, respectively [17, 22]. *Mp*ADC also has such spectral characteristics. The presence of absorption peak with a *λ*max at 337 and 423 nm indicated the association of PLP cofactor with *Mp*ADC [17, 22].

### Biochemical characterization of *Mp*ADC

**Fig. 3:**
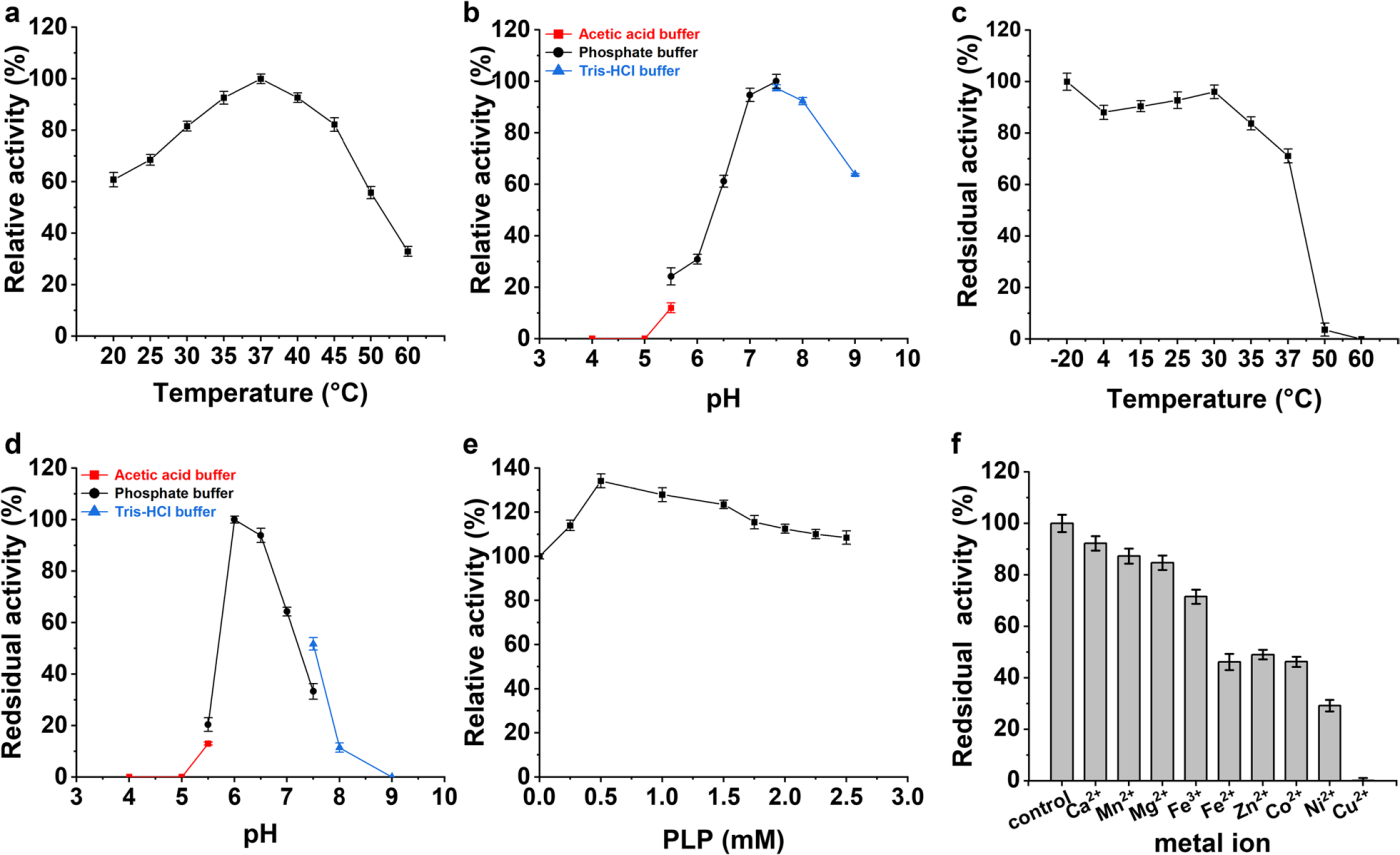
Biochemical characterization of *Mp*ADC. **a** Activity of *Mp*ADC at different reaction temperatures. The reaction was performed in buffer [0.2 M phosphate buffer (pH 7.5)] at different temperatures for 5 min. **b** Activity of *Mp*ADC at different reaction pHs. The reaction was performed in different buffers at 37 °C for 5 min. **c** Stability of *Mp*ADC at different temperatures. ADC activity was measured after treatment at the corresponding temperature for 12 h. **d** Stability of *Mp*ADC at different reaction pHs. The stability was analyzed by monitoring the residual activity after the enzyme was incubated in various pH conditions for 12 h at 30 °C. The maximum activity is defined as 100%. **e** Activity of *Mp*ADC at different concentration of PLP. The initial activity without PLP was set to 100%; **f** Effects of metal ions and chelating agents on its activity. The initial *Mp*ADC activity without metal ions and chelating agents was set to 100%. (All assays were performed in triplicate, and the standard deviations of the biological replicates were represented by error bars.)

*Mp*ADC showed considerable activity at temperatures range from 20 to 45 °C, with the best activity at 37 °C. It exhibited approximately 60% of its maximal activity at 20 °C and approximately 80% at 45 °C (Fig. 3a). The activity of *Mp*ADC is considerably high at pH from 6.5 to 8.0, but decreased sharply when the pH was lower than 6.5 or higher than 8.0 (Fig. 3b). *Mp*ADC showed good thermal stability, it kept over 80% activity after incubation at 4–35 °C for 12 h (Fig. 3c). It is stable at pH 6.0–to 7.0, but poorly stable at pH below 6.0 or above 7.0. It retained less than 50% of activity, after being incubated at pH below 6.0 or above 7.0 for 12 h. (Fig. 3d). *Mp*ADC belongs to the PLP-dependent enzyme family and requires PLP for the activation. To evaluate the effect of PLP concentration towards the enzyme activity, catalytic activities of *Mp*ADC were measured after adding PLP to different concentrations. It shows that *Mp*ADC exhibits good catalytic capability without extra PLP, which might owe to the activation of this enzyme by intracellular trace amounts of PLP. When PLP was added at a final concentration of 0.5 mM, the catalytic activity of *Mp*ADC was increased by 40%, but the higher concentration of PLP would inhibit the catalytic ability (Fig. 3e). All divalent metal ions exhibit inhibition of *Mp*ADC activity, suggesting that divalent ions adversely affect enzyme activity.

### Optimization of conditions for β-alanine production by whole-cell biotransformation

**Fig. 4:**
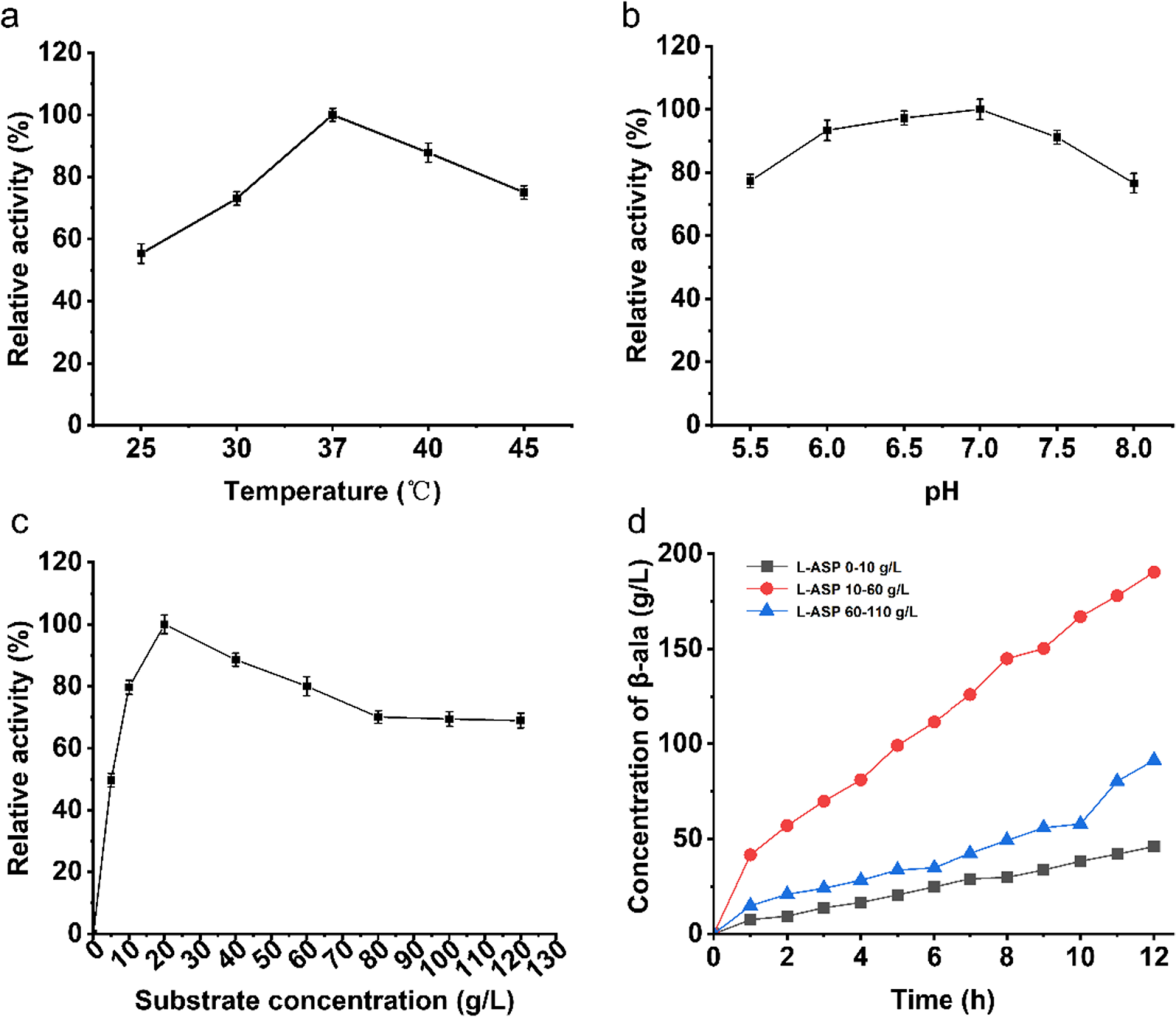
Production of β-alanine by whole-cell catalysis. **a** Activity of *Mp*ADC at different reaction temperatures; **b** Activity of *Mp*ADC at different reaction pHs; **c** β-alanine production at different substrate concentrations; the maximum yield was defined as 100%. **d** Whole-cell catalysis to produce β-alanine with L-ASP concentration of 0–10 g/L (black squares), 10–60 g/L (red dots) or 60–110 g/L (blue triangles)

Enzymes encased in the cells generally exhibit different properties compared to the free enzymes while they are surrounded by cytoplasm and have not undergone the damage by lysis process. In order to achieve the optimal whole-cell bioconversion efficiency, the effects of temperature, pH, and substrate concentration towards catalytic reaction using the whole cell expressing *Mp*ADC were also tested. As shown in Fig. 4a, the optimal temperature for whole-cell bioconversion of *Mp*ADC was 37 °C, which is similar with as that of the purified enzyme. However, unlike purified enzyme, the whole-cell expressed *Mp*ADC shows good catalytic activity in the pH range 5.5 to 8.0. This might owe to the buffer environment of the cytoplasm, cushioning the impact of pH from the external solution (Fig. 4b).

The whole cell expressing *Mp*ADC exhibited more than 80% catalytic activity under 10–60 g/L of L-ASP. With the concentration of L-ASP below 10 g/L, the catalytic activity increases as L-ASP increases. A high concentration of L-ASP seemingly inhibits the activity of the whole cell expressing *Mp*ADC while β-alanine production gradually goes down as the concentration of L-ASP increases over 60 g/L (Fig. 4c).

To further investigate the effects of L-ASP concentration towards the catalytic activity of the whole cell expressing *Mp*ADC, the time-dependent production of β-alanine was measured. As shown in Fig. 4d, when L-ASP concentration was kept at 0–10 g/L, the final β-alanine yield reaches 46.03 g/L after 12 h reaction. When L-ASP concentration was kept at 10–60 g/L, the maximum β-alanine production reaches 190.32 g/L after 12 h reaction. As a contrast, the production of β-alanine decreased to 91.44 g/L after 12 h incubation when L-ASP concentration was kept at 60–110 g/L. It is possible that too much L-ASP will result in “substrate inhibition”. This could imply that MpADC-dominated whole-cell biotransformation demands a decent substrate concentration.

### Structure prediction of *Mp*ADC and analysis

A precise stereological structure will help to understand the catalytic mechanism of enzymes and guide the protein engineering. The crystal structure of the insect ADC has not been resolved yet. Here, we predicted the *Mp*ADC structure by AlphaFold2, a machine learning approach with high accuracy [36]. In 2019, Liu et al*.* examined the state of aggregation of *Tc*ADC by gel filtration chromatography and concluded that the native *Tc*ADC is a dimer [23]. The results of sequence alignments (Fig. 1b) showed that *Mp*ADC shared 67.07% identity with *Tc*ADC. Thus, Alphafold2 predicted the structure of *Mp*ADC from the pattern of the dimer. The most reliable prediction structure was selected by predicted IDDT per position and estimated in SAVES v6.0. The Verify-3D result showed that the average 3D-1D score of 82.40% of the residues was ≥ 0.2, and the Ramachandran plot result showed the allowed region and the maximum allowed region other than alanine was greater than 95%. This demonstrated that the model conformed to the composite stereo conformation rules, and the relationship among the primary amino acid structure was good. Overall, the model quality is evaluated to be excellent, which could be used for the subsequent conservativeness and docking analysis.

**Fig. 5:**
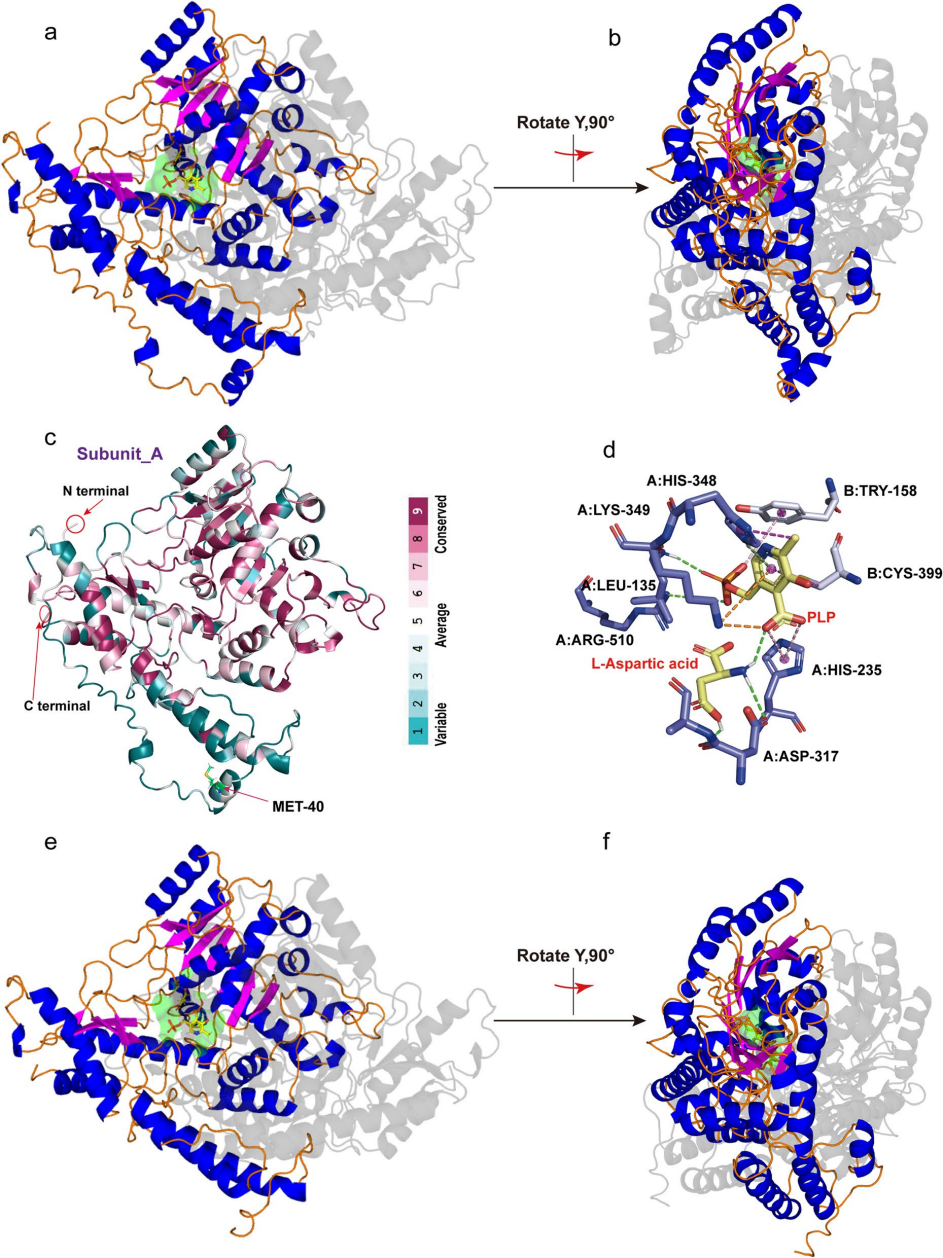
Three-dimensional structure analysis of *Mp*ADC. **a**, **b** Predicted 3D structure of *Mp*ADC. The predicted structure of subunit A was shown on the top (β-sheet, magenta; α-sheet, blue; loop, orange); the subunit B was shown at the bottom (grey). The predicted active pockets were shown in green. **c** The A-subunit of *Mp*ADC. The amino acids are colored by their conservation grades using the color-coding bar, with turquoise-through-maroon indicating variable-through-conserved. The C-terminal, N-terminal and 40th residue of N-terminal of A-subunit were indicated by red arrow. **d** Three-dimensional (3D) docking models between the substrates, PLP and L-ASP (light yellow sticks) and *Mp*ADC (the green dotted line, magenta dotted line, pink dotted line, orange dotted line and split pea represented hydrogen bonds, pi-alkyl interactions, pi-anion interactions, cation-pi interactions, pi-donor hydrogen bond, respectively). The predicted amino acid sites bound to the substrates include Leu135, His235, Asp317, His348, Lys349, and Arg510 from subunit A (purple sticks) and Tyr158 and Cys399 from subunit B (light-blue sticks). **e**, **f** The modeled 3D structure of variant Δ39. The spatial position corresponds to **a**

As shown in Fig. 5a, b, *Mp*ADC is a homodimer. The subunit of *Mp*ADC has a seven-stranded β-sheets nearly the N-terminal (β1, residues 189–193; β2, residues 226–231; β3, residues 252–255; β4, residues 284–288; β5, residues 313–317; β6, residues 342–345; β7, residues 358–362), a four-stranded β-sheets at the C-terminal (β8, residues 444–446; β9, residues 455–458; β10 residues 498–500; β11 residues 508–512), and 17 α-helices covering the outer side.

As shown in Fig. 1b, the amino acids involved in the activity as shown in other ADCs are also conserved in *Mp*ADC. While PLP reacted with Lys333 and Lys314, respectively, referring to the crystal structure of Q9Y600 (PDB ID: 2JIS), Q80WP8 (PDB ID: 6ENZ) [40, 41], we determined the amino acid sites reacted with PLP in *Mp*ADC and generated the molecular docking accordingly. The result of molecular docking is shown in Fig. 5d. The active pocket is located at the interface between the subunits and contains amino acid residues from both subunits (Fig. 5a, b). The active site engulfing the cofactor PLP and L-ASP are surrounded by several key conserved amino acid residues, that is instrumental to the decarboxylase reaction [43–45]. Lysine (Lys349) binds to PLP and histidine (His235) coordinates the carboxyl group of the substrates. The leaving carboxyl group, in a perpendicular position to the plane formed by the PLP ring and the Schiff base moiety, is potentially stabilized by Arg510 [35].

### Engineering *Mp*ADC by truncating its N-terminal

**Fig. 6:**
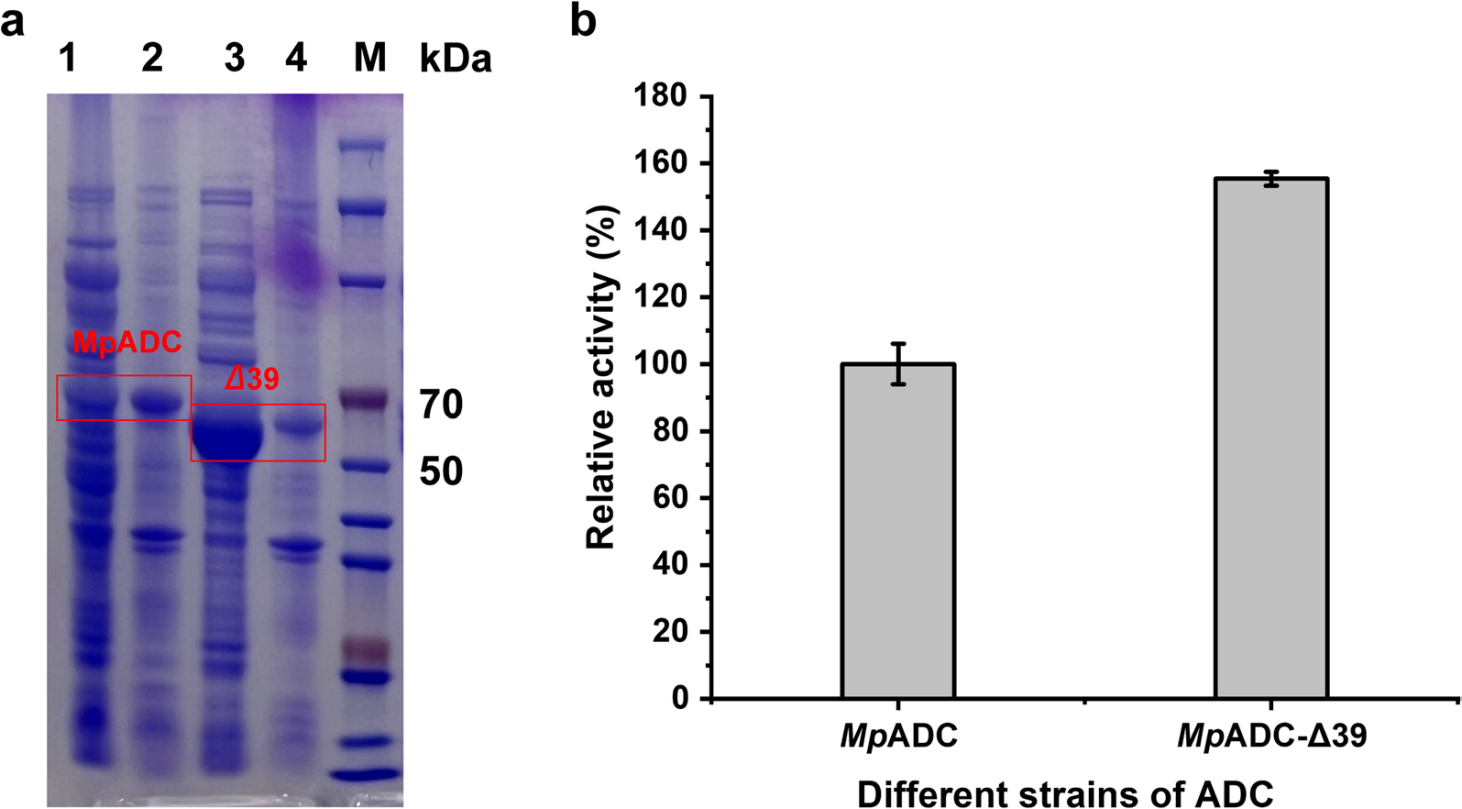
Expression and catalytic activity of *Mp*ADC-Δ39 variant. **a** Analysis of *Mp*ADC and *Mp*ADC-Δ39 expression by SDS-PAGE. M: protein marker; lanes 1 and 3: the supernatant of *E. coli* cells expressing *Mp*ADC and *Mp*ADC-Δ39; lanes 2and 4: the precipitation of *E. coli* cells expressing *Mp*ADC and *Mp*ADC-Δ39. **b** The relative activity of the cell expressing *Mp*ADC and *Mp*ADC-Δ39. Whole-cell catalysis was performed in a 1-mL reaction system containing 100 g/L fermented cells, 60 g/L L-ASP, 0.5 mM PLP (pH–6.5) on a magnetic stirrer (IKA® C-MAG HS 7) at 1500 rpm, 37 °C. After 5 min, the reaction was terminated by a 100 °C metal bath for 5 min. The experiment was performed in triplicate, and the standard deviations of the biological replicates were represented by error bars

As shown from sequence conservation analysis (Fig. 1b) and structure prediction (Fig. 5b), the N-terminal of *Mp*ADC is poorly conserved and seemingly drifted away from the core structure of *Mp*ADC. This N-terminal end polypeptide also seemingly disturbs the substrate to approach the active center, and might be redundant to the activity of *Mp*ADC. Predicting the structure of *Mp*ADC-Δ39 by AlphaFlod2 (Fig. 5e, f) suggests that the truncation of the N-terminal end of *Mp*ADC has no effect on the overall folding of the rest amino acids. Therefore, we experimentally constructed a variant Δ39, which, respectively, excised the peptide fragments from 1 to 39 amino acid residuals. Interestingly, SDS-PAGE analysis showed that the soluble expression of *Mp*ADC was obviously increased when the N-terminal peptide (1–39) was excised (Fig. 6a). The kinetics parameters of purified wild-type and mutant were tested. The *K*_m_ of *Mp*ADC and *Mp*ADC-Δ39 is 11.17 mM and 15.71 mM, respectively; whereas the *k*_cat_ value of *Mp*ADC and *Mp*ADC-Δ39 is 16.41 s^−1^ and 20.38 s^−1^, respectively. The *K*_cat_/*K*_m_ of *Mp*ADC and *Mp*ADC-Δ39 is 1.4 mM/s and 1.30 mM/s, respectively. Comparison of the kinetic parameters of mutant with wild-type, the substrate affinity of the mutant decreases, but the conversion efficiency increases. In general, although the purified variant and wild-type enzyme showed similar catalytic efficiency, overall activity of cell expressing *Mp*ADC-Δ39 was 55.41% higher than that of its wild type due to increased soluble expression (Fig. 6b).

### Production of β-alanine using fermented cells expressing *Mp*ADC-∆39

**Fig. 7:**
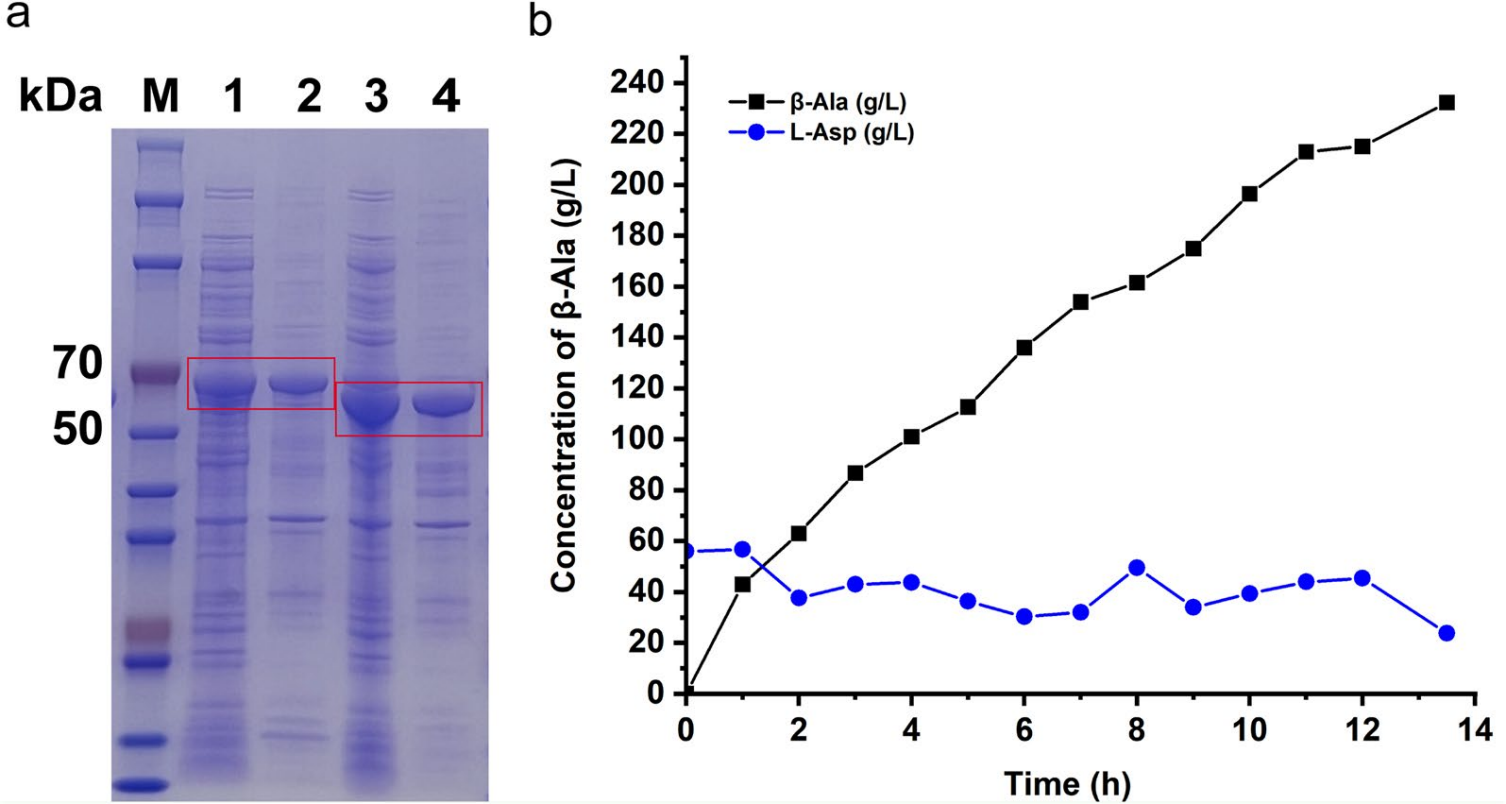
Production of β-alanine by whole-cell catalysis. **a** SDS-PAGE analysis of *Mp*ADC and *Mp*ADC-Δ39 expressions in *E. coli* cells cultured from a 15 L fermentor. M: protein marker; lanes 1, 3: the supernatant of fermented *E. coli* cells expressing *Mp*ADC and *Mp*ADC-Δ39; lanes 2, 4: the precipitation of fermented *E. coli* cells expressing *Mp*ADC and *Mp*ADC-Δ39, respectively. **b** The 1-L reaction system consists of 50 g/L cells (corresponding OD_600_ = 18.4), 55 g/L L-ASP and 0.5 mM PLP. The pH was maintained at about 6.3–6.5 by adding L-ASP

In order to construct a whole-cell bio-transforming process that can be scaled up for industrial production of β-alanine, we tested the expression of *Mp*ADC and its derivatives using a 15-L fermentor. Both of them were expressed with pGrO7 in *E. coli*. Different from that in the shaker, the wild-type *Mp*ADC was expressed essentially as an inclusion body in the 15-L fermenter (Fig. 7a). In contrast, the *Mp*ADC-∆39 variant still maintained good soluble expression in 15-L fermenters (Fig. 7a).

Fermented cells were harvested and used in alanine synthesis reactions. The 1-L reaction system consists of 50 g/L cells expressing *Mp*ADC-∆39 (corresponding OD_600_ = 18.4), 55 g/L L-ASP and 0.5 mM PLP. The pH was maintained at about 6.3–6.5 by adding L-ASP. As shown in Fig. 7b, after 14 h of reaction, the final concentration of β-alanine reached 232.36 g/L and the residual concentration of L-ASP was 23.83 g/L, the conversion rate was determined to be 94.68% and the average space–time yield of β-alanine was 17.21 g/L/h.

## Discussion and conclusion

The whole-cell catalysis by ADC enzyme is an important method for the production of β-alanine in an environment-friendly manner. Despite studies that have been done for several decades, no strategy was commercialized due to low yield, and correspondingly high cost. The prokaryote ADCs have substrate-dependent inactivation effects, thus are not suitable for large-scale industrial applications [11, 21, 46]. Several studies have reported that eukaryote ADCs exhibited industrial application potential [17, 47]. For example, the whole-cell catalysis using sulfinic acid decarboxylase and its derivatives from *T. castaneum* showed a high β-alanine production yield. Using cells expressing G369A mutant with OD_600_ of 200 of *Tc*ADC for whole-cell catalysis, up to 162 g/L β-alanine was produced in 35 h [23]. Nevertheless, the high density of cells invested into the whole-cell catalysis reaction might result in high fermentation and complex production extract process. Therefore, it is controversial whether this strategy can be utilized for further large-scale application. The cysteine sulfuric acid decarboxylase from *M. persicae* we presented here showed the highest β-alanine production rate, to date. The investment of *E. coli* cells expressing *Mp*ADC mutant with OD_600_ of 18.4 from 15 L batch fermentation into whole-cell catalysis reaction, up to 232.36 g/L β-alanine could be produced in 13.5 h, which gets more yield with less time and fewer amount of cell **(**Table 1). The reduction of cell investment should also decrease the cost of fermentation, energy consumption and pollution load in the fermentation process. Moreover, while the cell amount was decent and the reaction system was relatively easy, this strategy should be easy to follow for large scaled catalysis reaction. Therefore, the *Mp*ADC-based strategy presented here should be very useful for the environmental friendly production of β-alanine.

**Table 1:** Comparison of β-alanine production by ADCs from different sources

Source	Enzymes	The concentration of cells^2^	Reaction time (h)	yield (g/L)	Space–time yield (g/(L*h))	References
**Prokaryotic**	**panD**					
*C. glutamicum*	*Cg*ADC	20 g/L	36	12.85	0.357	[12]
*B. subtilis*	I46V/I88M/K104S/I126*	20 g/L	14	124.3	9.563	[21]
*B. subtilis*	E56S	OD_600_ = 100	9	215.3	23.90	[11]
*B. aryabhattai*	I88M	20 g/L	12	128.67	10.72	[26]
**Eukaryotic**	**ADC**					
*T. castaneum*	R98H/K305S	OD_600_ = 100	48	170.5	3.550	[8]
*T. castaneum*	G369A	OD_600_ = 200	35	162	4.629	[23]
*T. castaneum*	K221R	OD_600_ = 200	23	134.72	5.857	[49]
*M. persicae*	Δ39	50 g/L(OD_600_ = 18.4)	13.5	232.36	17.21	This work
**Co-expressing PanD and ADC**						
*B. subtilis*/*T. castaneum*	BTW	OD_600_ = 60	30	271.5	9.05	[48]

1. ‘*’ represents the termination codon

2. The amount of engineered bacteria added was expressed as the concentration of bacterial suspension (OD_600_ or the concentration of bacterial wet weight)

β-Alanine is involved in many physiological processes in insects, including melanin deposition, cuticle hardening, and circadian rhythm regulation [28]. The main pathway for β-alanine synthesis in insects is α-decarboxylation of aspartate, but the enzymes involved in this process are less well understood. Li et al*.* expressed two putative GDC-like enzymes from mosquitoes and demonstrated that one of them was ADC, providing the first evidence of specific ADC in mosquitoes [22]. Insects ADCs have high sequence identity with mammalian CSADCs and GAD [17, 22, 28]. However, prior to our investigation, only three ADCs from insects, derived from *T. castaneum* (*Tc*ADC), *D. melanogaster* (*Dm*ADC), and *A. aegypti* (*Ae*ADC), had been validated. Despite having a very modest level of expression and activity, *TcADC* has been found to catalyze the synthesis of β-alanine from aspartate with a substantially greater specific activity than ADCs from prokaryotes. As shown in our study, the *Mp*ADC shared 66.67, 65.03% and 62.11% towards *Tc*ADC, *Ae*ADC and *Dm*ADC. The amino acids involved in the activity as shown in other ADCs are also conserved in *Mp*ADC. The effective expression and characterization of *Mp*ADC in our study should lead to a better understanding of ADCs from insects.

So far, the bacteria originated ADCs have been well characterized. However, they generally contain self-shearing activity and are therefore not suitable for practical industrial application. Previous study indicated that the *E. coli* ADC, PanD formed a tetramer and contained three previously sheared π-proteins and one un-sheared π-protein [50]. Through homology modeling, molecular docking, and comparison and analysis with the crystal structures of the resolved enzyme with high homology, we reached the following conclusions. In contrast to PanD, the 3D structure and spectral absorption characteristic of *Mp*ADC illustrated that it has typical characteristics of the PLP-dependent ADCs. It formed a homodimer and each subunit contains a PLP targeted Lys residue near the C-terminal (Lys349), which formed the active pocket between the two subunit interfaces. Whole-cell catalysis using the expression of *Mp*ADC and its derivatives in *E. coli* has shown that its activity can be sustained over a long incubation period. Overall, these results suggested that *Mp*ADC should not have post-cutting activity, which enabled it to be more robust in practical industrial applications. In addition, it is worth mentioned that the N-terminal end of *Mp*ADC is indeed an interesting structure. The 3D structural modeling of *Tc*ADC and *Ae*ADC also revealed the existence of an unconventional peptide at the N-terminal (data not shown). Although it remained unknown why the N-terminal was present in such ADCs in nature, the truncation of the N-terminal end of *Mp*ADC does improve its expression. This might provide a good reference for investigating the structural function of other similar ADCs.

**Fig. 8:**
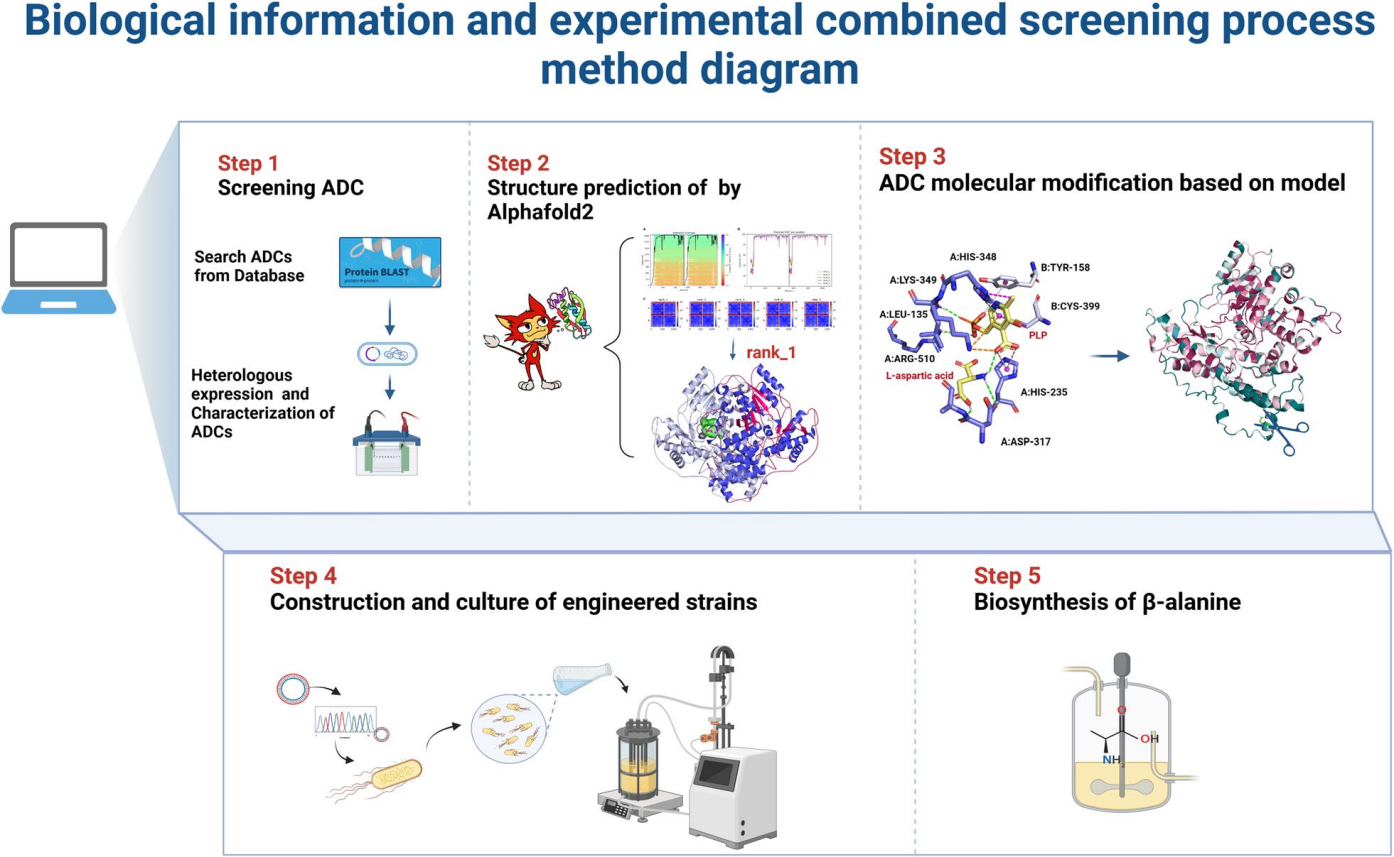
Biological information and experimental combined screening process method diagram

Finally, in this study, we found a β-alanine production strategy by the combination of bioinformatics analysis and experimental optimization. Firstly, homology sequence analysis was utilized to find ADC enzyme candidates from eukaryotes. Then, whole-cell catalysis using *E. coli* cells expressing these enzymes was used to screen the target *Mp*ADC. Next, expression optimization was performed by gene-based codon optimization and chaperon protein-based host optimization. The high-scored structural prediction by Alphafold2 and a detailed sequence conservation analysis identified an N-terminal unique region of *Mp*ADC. Structural predictions show that the N-terminal excision does not disrupt the overall structure of the enzyme. Further, engineering of the *Mp*ADC by truncation of its N-terminal region resulted in significantly improved expression level. Finally, catalysis optimization enabled 15 L fermented cells expressing *Mp*ADC-∆39 mutant showed the best β-alanine production yield. This should be an “easy to follow” approach for screening enzymes used for another whole-cell catalysis industrial application. Although our research took for a relatively long time as the first “method” exploration, this approach should ideally be performed within 2 months. Moreover, if automation equipment was applied, the speed would be faster. Overall, the “IT (information technology)” and “BT (biotechnology)” based strategy presented here should provide a very good reference for future whole-cell catalysis strategy screening (Fig. 8).

In conclusion, we identified and characterized a l-aspartate-α-decarboxylase, *Mp*ADC, from an aphid, *M. persicae,* based on “bioinformatic analysis” and “experimental validation”. Through established process of whole-cell enzymatic catalysis with *E. coli* cell expressing *Mp*ADC-∆39, the average β-alanine yield reaches 17.22 g/L/h, which represents the highest yield, found so far. Our study should provide a very good basis for the industrial production of β-alanine by whole-cell enzymatic catalysis, and a good reference for exploring whole-cell enzymatic catalysis avenues for other compounds.

## Data Availability

All data generated or analyzed during this are included in this published article.
